# HPV Status and Individual Characteristics of Human Papillomavirus Infection as Predictors for Clinical Outcome of Locally Advanced Cervical Cancer

**DOI:** 10.3390/jpm11060479

**Published:** 2021-05-27

**Authors:** Liana Mkrtchian, Irina Zamulaeva, Liudmila Krikunova, Valentina Kiseleva, Olga Matchuk, Liubov Liubina, Gunel Kulieva, Sergey Ivanov, Andrey Kaprin

**Affiliations:** 1 A. Tsyb Medical Radiological Research Center—Branch of the National Medical Research Radiological Center of the Ministry of Health of the Russian Federation, Korolev Str.-4, 249036 Obninsk, Russia; zamulaeva@mail.ru (I.Z.); gynec@mrrc.obninsk.ru (L.K.); kivapim@mrrc.obninsk.ru (V.K.); matchyk@mail.ru (O.M.); lyubina-57@mail.ru (L.L.); gunelka2010@yandex.ru (G.K.); ivanov@mrrc.obninsk.ru (S.I.); 2 National Medical Research Radiological Center of the Ministry of Health of the Russian Federation, Korolev Str.-4, 249036 Obninsk, Russia; mrrc@mrrc.obninsk.ru

**Keywords:** cervical cancer, radiotherapy, chemoradiotherapy, prognosis, human papillomavirus

## Abstract

This study is aimed at searching for an informative predictor of the clinical outcome of cervical cancer (CC) patients. The study included 135 patients with locally advanced cervical cancer (FIGO stage II–III) associated with human papillomavirus (HPV) 16/18 types or negative status of HPV infection. Using logistic regression, we analyzed the influence of the treatment method, clinical and morphological characteristics, and the molecular genetic parameters of HPV on the disease free survival (DFS) of patients treated with radiotherapy or chemoradiotherapy. Multivariate analysis revealed three factors that have prognostic significance for DFS, i.e., HPV-related biomarker (HPV-negativity or HPV DNA integration into the cell genome) (OR = 9.67, *p* = 1.2 × 10^−4^), stage of the disease (OR = 4.69, *p* = 0.001) and age (OR = 0.61, *p* = 0.025). The predictive model has a high statistical significance (*p* = 5.0 × 10^−8^; Nagelkirk’s R^2^ = 0.336), as well as sensitivity (Se = 0.74) and specificity (Sp = 0.75). Thus, simultaneous accounting for the clinical and molecular genetic predictors (stage of the disease, patient age and HPV-related biomarker) makes it possible to effectively differentiate patients with prognostically favorable and unfavorable outcome of the disease.

## 1. Introduction

Cervical cancer (CC) continues to occupy one of the leading places in the morbidity and mortality of young women [1]. Despite the widespread implementation of screening programs, there is quite a high proportion of locally advanced CC, the treatment efficiency of which does not exceed 60% in some countries. The search for an informative predictor of unfavorable clinical outcome of CC can play a decisive role in the optimal planning of treatment for locally advanced forms of the disease.

The clinical studies revealed the main factors influencing the effectiveness of CC treatment: degree of spread, form of growth, morphological structure of tumor, patient’s age, etc. [2]. At the same time, it turned out that the treatment effectiveness can vary greatly between patients with the same clinical and morphological characteristics. Therefore, the search for new prognostic biomarkers of the effectiveness of CC treatment remains relevant to this day [3,4].

The individual characteristics of human papillomaviruses (HPV) of high carcinogenic risk are among the most attractive prognostic markers because of the proven ability of the virus to modulate the sensitivity of tumor cells to various impacts [5,6]. It is known that the protein products of viral oncogenes E6/E7 are capable of inactivating cellular proteins p53 and pRb and thereby disrupting the mechanisms of apoptosis and cell cycle control, which contributes to an increase in the resistance of tumor cells to damaging agents [7,8,9,10]. In addition, it has been shown that E6 oncoprotein increases the expression of nucleotide excision repair gene ERCC1, which also leads to an increase in the resistance of tumor cells to ionizing radiation and alkylating agents [11]. On the other hand, it was found that oncoprotein E6 can increase apoptotic cell death induced by anticancer drugs [12] and ionizing radiation [13], for example, by increasing the expression and functional activity of Cdc2, which plays an important role in the cell cycle control. Overall, viral proteins can modify a number of intracellular biochemical signaling pathways that control cellular responses, especially cell proliferation [7,14] which is one of the most important characteristics of malignant neoplasms, affecting their aggressiveness and the effectiveness of CC treatment. 

The integration of HPV DNA into the cell genome is predominantly associated with disruption of the E2 gene open reading frame, which leads to the loss of E2 functional activity as a negative regulator of the transcription of viral E6/E7 oncogenes and, as a result, to an increase in the expression of E6 and E7 oncoproteins, although the number of viral DNA copies decreases [15,16]. In turn, an increase in the expression of these proteins can change the sensitivity of CC to chemo- and radiotherapy.

Another important mechanism of HPV influence on the chemo- and radiosensitivity of tumor cells is associated with the effect of HPV on the pool of cancer stem cells (CSC), which are resistant to many anticancer agents in comparison with the rest of the tumor cells [17,18]. The results of several studies suggest that there is a relationship between HPV and the formation of the CSC pool, as well as with the CSC response to antitumor impacts [19,20,21,22,23].

Taken together, these data, obtained mainly in vitro, indicate the participation of HPV in the modulation of radio- and chemosensitivity of tumor cells. At the same time, the role of HPV in vivo may be somewhat different, taking into account the influence of numerous microenvironmental factors on the sensitivity of malignant neoplasms to anticancer agents. These factors include physicochemical characteristics (hypoxia, pH of the extracellular medium) and many signaling molecules (for example, TGF-b1, FGF, IL-6, HIF, Wnt ligands, etc.) secreted by not only tumor cells but also various stromal cells, including endothelial, immune cells, tumor-associated macrophages, fibroblasts, and normal stem cells. 

Indeed, the results of studying the relationship between various HPV parameters and clinical outcome in patients with CC after radiation and/or chemoradiation therapy are controversial. The inconsistency in the assessment of the prognostic value refers to such HPV parameters as genotype [24,25,26,27,28], viral load (VL) [29,30,31,32,33] and the integration of HPV DNA into the cell genome [34,35,36,37]. At the same time, the presence of HPV before treatment is almost unambiguously considered as a favorable prognostic marker, and HPV-negativity is associated with an increased risk of disease progression after treatment [38,39].

The purpose of this study was to clarify the prognostic value of such parameters of HPV infection as presence/absence of high risk HPV DNA (HPV status), its genotype, VL, and the integration of HPV DNA into the cell genome in combination with traditional clinical and morphological indicators (age, stage, histological type, nuclear grade, form of tumor growth, infiltration of parametrium, metastases to lymph nodes).

## 2. Materials and Methods

### 2.1. Patients

The HPV status and genotype were determined in tumor material of 173 patients with CC stages II–III according to the classification developed by the International Federation of Gynecology and Obstetrics (FIGO). Patients were treated at the Department of Radiation and Combined Methods for the Treatment of Gynecological Diseases, A. Tsyb Medical Radiological Research Center (MRRC, Obninsk, Russian Federation). This study was approved by the Ethics Committee of MRRC, informed consent was obtained from all patients for the study. One hundred thirty-five (out of 173) patients with HPV-negative or HPV16/18-positive tumors were selected for follow-up study of prognostic value of the HPV status, parameters of HPV infection, clinical and morphological indicators. The diagnosis was morphologically verified in all patients. The histological type of tumor was identified in accordance with the WHO classification [40]. Patients underwent a complete clinical and laboratory examination (bimanual rectovaginal examination, magnetic resonance imaging (MRI) and/or computed tomography (CT) of the pelvic organs and abdominal cavity, lung radiography, etc.). Based on the data obtained, staging of the disease was carried out in accordance with the FIGO recommendations and the TNM classification [41,42].

The effectiveness of CC patient treatment was assessed by disease-free survival (DFS) according to the criteria for the occurrence of loco-regional relapses and distant metastases based on the annual clinical and radiological examination using rectovaginal examination, ultrasound, computed and/or magnetic resonance imaging in accordance with the RECIST v. 1.1 (Response Evaluation Criteria in Solid Tumors) [43]. An unfavorable clinical outcome of the disease was considered to be a progression, including loco-regional relapses and/or distant metastases, or death due to disease progression. The average follow-up period was 33.2 ± 19.6 months, maximum of 60 months.

### 2.2. Polymerase Chain Reaction (PCR)

The presence of HPV DNA of 14 high risk genotypes (16, 18, 31, 33, 35, 39, 45, 51, 52, 56, 58, 59, 66, 68) was determined in united scrapings from endo- and exocervix of 173 CC patients before treatment. Real-time PCR on “Rotor-Gene” (Corbett Research, Sydney, Australia) was performed using the test-systems “AmpliSens HPV HCR screen-titer-FL” and “AmpliSens HPV HCR genotype-titer FL” (Central Research Institute of Epidemiology, Moscow, Russia).

The physical state (episomal or integrated form) of the viral DNA was assessed in HPV16- and HPV18-positive patients using kits containing primers and probes designed to specifically amplify the E2 and E7 genes of these viruses (Central Research Institute of Epidemiology, Moscow, Russia). This limitation is due to the fact that the set of reagents at our disposal allowed us to assess the degree of integration of HPV DNA 16 and 18 types only. Sites of HPV16 or HPV18 E2 and E7 genes and human β-globin gene were amplified in one tube in triplicate for each clinical sample. Standard samples with known concentration of HPV16 or HPV18 DNA were amplified in each experiment. The number of genomic equivalents of E2 and E7 was calculated from the calibration curves obtained on these standard samples. The quantitative load of HPV DNA was expressed in logarithms of E7 genomic equivalents, normalized to 200,000 genomic equivalents of β-globin or 100 thousand cells. The degree of HPV DNA integration was assessed by the ratio of E2 and E7 genomic equivalents, based on the fact that the E7 gene remains intact during the integration of viral DNA into the cell genome; therefore, the number of its copies in both forms of viral DNA (episomal and integrated) is the same. On the contrary, the E2 gene, as a rule, is destroyed and the number of its copies decreases. The degree of HPV DNA integration was calculated by the formula (1 − E2/E7) × 100%, where E2 and E7 are the number of genomic equivalents of the corresponding genes. The absence of amplification signal for E2 gene in the presence of such signal for E7 gene corresponds to 100% integration of viral DNA into the cell genome.

Data on VL in HPV 16/18 positive patients were interpreted in accordance with the following criteria:

- The number of E7 gene copies is less than 10^3^ per 100 thousand cells (lgE7 < 3)—low viral load;
- The number of E7 gene copies is equal to or more than 10^3^, but less than 10^5^ per 100 thousand cells (3 ≤ lgE7 < 5)—moderate viral load;
- The number of E7 gene copies is equal to or more than 10^5^ per 100 thousand cells (lgE7 ≥ 5)—high viral load.

### 2.3. Radiation and Chemoradiation Therapy

**Figure 1:**
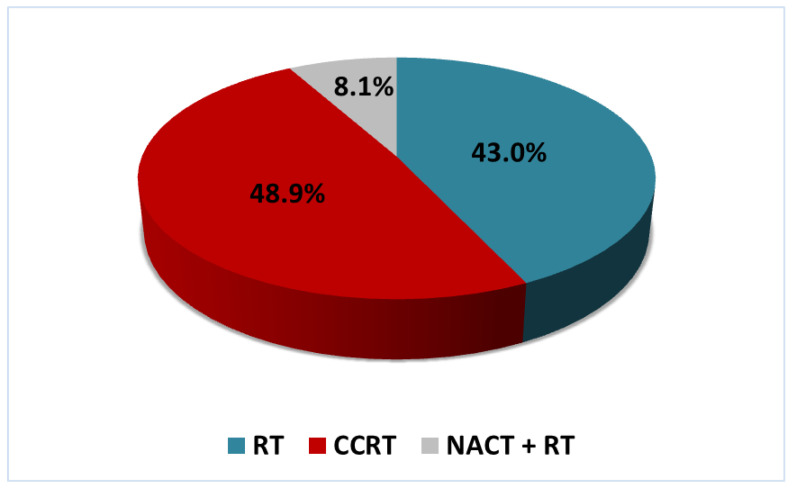
Distribution of 135 patients with cervical cancer (CC) by the treatment methods: radiation therapy (RT), concurrent chemoradiation therapy (CCRT) or neoadjuvant chemotherapy followed by radiotherapy (NACT + RT).

Patients underwent radical courses of radiation therapy (RT) in conventional regimen, concurrent chemoradiation therapy (CCRT) or neoadjuvant chemotherapy followed by radiotherapy (NACT + RT) (Figure 1).

RT included external beam irradiation of primary tumor focus and areas of regional metastasis on linear electron accelerator SL-75-5 (Philips, Guildford, UK) with photon radiation energy of 6 MeV at a single dose of 2.0 Gy daily on working days up to a total dose (TD) of 30.0 Gy. Then, intracavitary irradiation was performed on brachytherapy apparatus with sources of ^60^Co high activity at a single dose of 5.0 Gy two times per week to a TD of 50.0 Gy. TDs for the full course of RT were: primary focus 75.0–82.0 Gy, areas of regional metastasis 58.0–62.0 Gy, bladder 45.0–50.0 Gy, rectum 45.0–50.0 Gy. 

CCRT included RT during polychemotherapy (cisplatin 20 mg/m^2^ and 5-fluorouracil 200 mg/m^2^ on days 1–5) which was started simultaneously with external beam irradiation and performed in 2–3 cycles with an interval of 21 days.

NACT + RT included preradiation polychemotherapy (cisplatin 50 mg/m^2^ for 1 day, topotecan 0.75 mg/m^2^ for 1–3 days, 2–3 cycles with an interval of 21 days) and a radical course of RT, which was started 7–10 days after first cycle of polychemotherapy.

### 2.4. Statistical Analysis

Statistical processing of data was carried out using Software Package Statistica 10.0 (StatSoft, Inc., Minneapolis, MN, USA), SPSS Statistics 23.0 (International Business Machines Corp., Armonk, NY, USA), MedCalc 13.3.3.0 (MedCalc Software Ltd., Ostend, Belgium). For descriptive statistics, means and standard error (SE) were used. We calculated odds ratio (OR) of unfavorable outcome of the disease with 95% confidence interval (CI), sensitivity, specificity and accuracy (area under curve—AUC) of the prognostic test with Receiver Operating Characteristic (ROC) analysis.

The significance of differences for bivariate predictors was assessed using Fisher’s exact test. If the number of possible predictor values was more than two, a univariate logistic regression was constructed; significance was assessed by Wald test. Life table and Kaplan–Meier methods were used to assess DFS for various periods of observation. Log rank test was used to compare survival between groups and determine level of significance. Multivariate analysis was performed using multiple logistic regression. Hazard ratio (HR) was assessed with Cox proportional hazard model. A *p* values < 0.05 were considered statistically significant.

## 3. Results

### 3.1. Prevalence and Molecular Genetic Parameters of HPV

High risk HPV DNA was not detected in 20 out of 173 (11.5%) patients. One hundred and fifteen (66.5%) persons turned out to be HPV16/18-infected, including 95 HPV16-positive and 20 HPV18-positive cases. One patient was found to have simultaneously HPV 16 and 18 types; however, the case was assigned to the group of patients with HPV 16 due to the higher VL of type 16 (6.61) compared to type 18 (1.17). Other HPV genotypes (31, 33, 35, 39, 45, 51.52, 56, 58 and 59) were found in the remaining 38 (22.0%) patients. Samples from the last 38 patients were excluded from further research due to methodological limitations in determining the degree of integration of viral DNA into the cell genome.

Thus, the study group consisted of 135 patients with HPV-negative or HPV 16/18 positive tumors. The clinical and morphological characteristics of the patients are presented in Table 1. The average age of the patients was 48.8 ± 11.8 years. Squamous cell carcinoma of various degrees of differentiation was found in the vast majority (88.1%) of patients, adenocarcinoma or adenocarcinoma with mixed subtypes—in 11.9% of patients. According to the anatomical extent of the disease, the patients were distributed almost evenly: stage II of the disease according to FIGO was diagnosed in 62 individuals (45.9%), stage III—in 73 (54.1%). Metastatic lesions of regional lymph nodes were detected in 65 (63.0%) patients.

Molecular genetic parameters of HPV in tumors of 115 HPV16/18-positive patients are shown in Table 2. One hundred and two patients (88.7%) had one genotype of the virus (mono infection). Infection with several types of high risk HPV (multiple infection) was detected in 13 (11.3%) HPV16-infected patients, of which 10 (8.7%) individuals had 2 HPV genotypes, 3 (2.6%) individuals had 3 genotypes, including the combinations of HPV16 with 18, 31, 45, 59, 68 genotypes.

High VL (on average, lgE7 = 6.6 ± 1.3) was observed most often, namely in 88 (76.5%) patients. VL was moderate (on average, lgE7 = 4.3 ± 0.5) in 23 (20.0%) patients, and low VL (on average, lgE7 = 1.4 ± 1.1) was found only in 4 (3.5%) patients.

The presence of HPV 16/18 DNA integration of various degrees into the cell genome (integrated form) was found in the majority of patients—in 66 cases (57.4%), of which 26 cases had complete (100%) integration of viral DNA. Absence of integration (episomal form) was registered in 49 (42.6%) patients.

VL and physical state of viral DNA (integrated or episomal form) were not significantly different in patients with stages II and III of the disease (*p* > 0.05 according to Fisher’s test).

**Figure 2:**
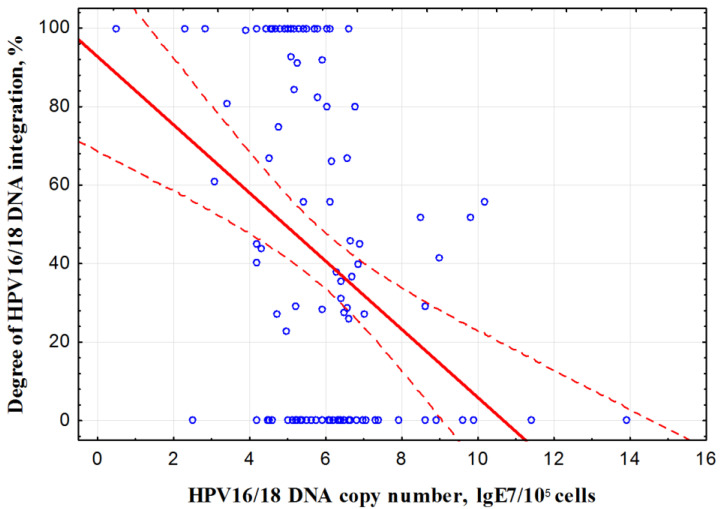
Correlation of molecular genetic parameters of human papillomavirus (HPV)16/18 (*n* = 115). *Y*-axis: 0%—no integration of HPV DNA into the host cell genome (episomal form), 100%—complete integration.

A comparative study of VL and the degree of viral DNA integration revealed an inverse linear correlation (Spearman correlation coefficient *r* = −0.39, *p* = 0.00002) (Figure 2).

### 3.2. Univariate Analysis of Clinical Outcome According to Candidate Predictor Variables

In univariate analysis, the clinical outcome of the disease depended on the HPV status and physical state of HPV16/18 DNA (episomal or integrated form) (Table 3). Thus, the probability of an unfavorable outcome was significantly higher in HPV-negative than in HPV 16/18-positive patients (*p* = 0.018; OR = 3.31). Interestingly, the probability of unfavorable outcome in HPV-negative patients was comparable to that observed for cases of HPV DNA integration (*p* = 0.310; OR = 1.76). On this basis, cases with integrated form of HPV 16/18 DNA and HPV-negative cases were pooled in a multivariate analysis.

The presence of HPV 16/18 in episomal form significantly increased the probability of favorable outcome (*p* = 4.7 × 10^−5^; OR = 3.66).

No dependence of the clinical outcome on the genotype, the number of genotypes, and the level of VL was found.

Of the clinical and morphological indicators, only stage (*p* = 0.001; OR = 3.89), histological type (*p* = 0.035; OR = 2.32) and parametrial infiltration (*p* = 0.020; OR = 2.77) were associated with the clinical outcome of the disease.

**Figure 3:**
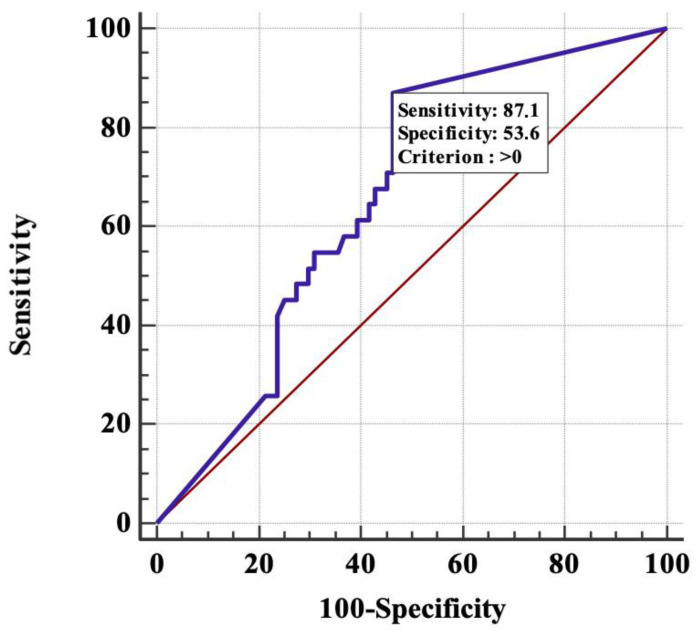
ROC-curve, built on the basis of data on the degree of integration of HPV 16/18 DNA into the cell genome (from 0% in the absence of integration to 100% in the case of complete integration), to select optimal discriminator that separates patients into groups with relatively favorable and unfavorable clinical outcome of the disease.

Taking into account the data on the association of HPV DNA integration into the cell genome with unfavorable clinical outcome, it was interesting to find the optimal discriminatory level of integration degree, dividing patients into groups with a favorable and unfavorable clinical outcome. It was found by the ROC analysis that such a discriminator is the presence/absence of HPV16/18 DNA integration into the host cell genome regardless of the degree of integration (Figure 3). The AUC value was 0.7 (95% CI, 0.6–0.8; *p* = 0.0016) for a period of 5 years.

The study results served as the basis for combining patients with integrated HPV16/18 DNA (regardless of the degree of integration) and HPV-negative patients into one prognostic group. As a result of this combining, a single biomarker including HPV-negativity or presence of HPV16/18 DNA integration into the cell genome was created. In the general group of CC patients with stages II–III, OR of unfavorable outcome was several times higher in patients with presence of the biomarker than in other patients, and reached 8.9 (95% CI, 2.9–27.6; *p* = 0.0001) for period of 5 years. Separately for stage III, OR was 5.8 (95% CI, 1.7–19.9; *p* = 0.0023). OR for stage II was not calculated, since all patients under observation with episomal form of HPV 16/18 DNA were alive for 5 years without disease progression.

### 3.3. Kaplan–Meier Analysis of Disease Free Survival

In the general group of CC patients with stages II–III, DFS did not differ significantly between patients with different levels of VL at all periods of observation. The same data were obtained as a result of separate analysis for stages II or III of the disease. Thus, 5-year DFS of patients with stage II was 100% at low VL, 65.0 ± 14.9% at moderate VL, and 81.7 ± 7.4% at high VL (*p* = 0.067 according to Log Rank test); with stage III–50.0 ± 35.3%, 36.4 ± 20.2% and 47.3 ± 9.4%, respectively (*p* = 0.864).

**Figure 4:**
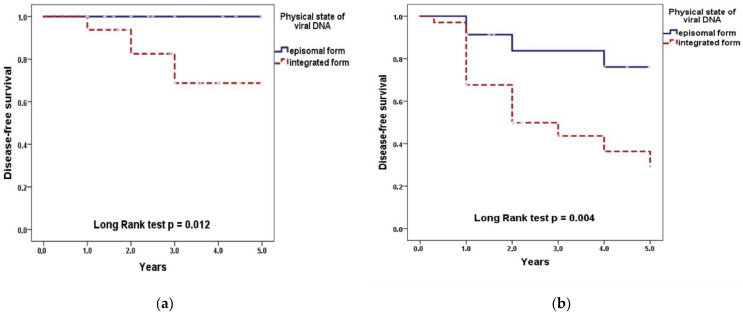
Disease free survival of CC patients with stages II (**a**) and III (**b**) depending on the physical state of the viral genome (episomal or integrated form).

The significant decrease in DFS was found in patients with an integrated form of HPV 16/18 DNA (regardless of the integration degree) compared with an episomal form: 48.8 ± 7.6% versus 88.5 ± 5.6% for all periods of observation (*p* = 0.0002 according to Log Rank test). At stage II of the disease, the 5-year DFS of patients with episomal form was 100%, with integrated form—68.8 ± 9.4% (*p* = 0.012); at stage III of the disease—76.1 ± 11.0% and 29.1 ± 10.6%, respectively (*p* = 0.004) (Figure 4).

Patients with HPV-negative CC showed low 5-year DFS (50.3 ± 35.3%; at stage II and 48.5 ± 13.1% at stage III), similar to that in HPV 16/18—positive patients with integrated form (*p* = 0.58 and *p* = 0.96, respectively).

**Figure 5:**
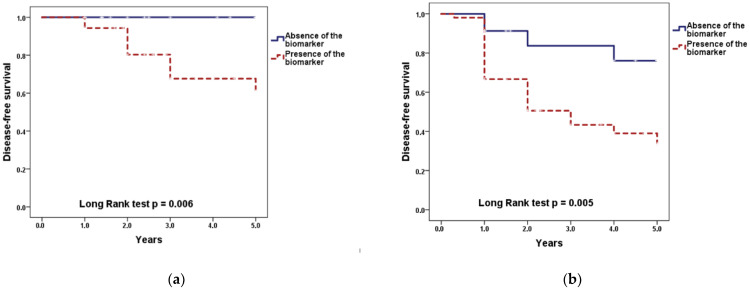
Disease free survival of CC patients with stages II (**a**) and III (**b**) depending on the absence/presence of the biomarker (HPV-negativity or integration of HPV16/18 DNA into the cell genome).

The 5-year DFS of CC patients with stages II–III in the prognostically favorable group (absence of the biomarker) was significantly higher than in the prognostically unfavorable group (presence of the biomarker): 88.8 ± 5.6% versus 44.8 ± 6.8% (*p* = 4.55 × 10^−5^ according to LogRank test), while with stage II-100% and 60.9 ± 10.4%, respectively (*p* = 0.006), with stage III-76.1 ± 11.0% and 33.4 ± 8.7%, respectively (*p* = 0.005) (Figure 5).

### 3.4. Multivariate Analysis of Clinical Outcome

Multivariate analysis included all variables regardless of statistical significance on univariate analysis: clinical and morphological characteristics (age, FIGO stage, histological type, nuclear grade, form of tumor growth, parametrial infiltration, metastases in lymph nodes, treatment method) and molecular genetic parameters of HPV infection (presence/absence of the biomarker, HPV genotype, number of genotypes, VL). As a result of multivariate analysis, three independent predictors of clinical outcome of locally advanced CC were identified: presence/absence of the biomarker (HPV-negative status or HPV16/18 DNA integration) (*p* = 1.2 × 10^−4^; OR = 9.67), stage of the disease (*p* = 0.001; OR = 4.69), age (*p* = 0.025; OR = 0.61) (Table 4).

Thus, the results of multivariate analysis showed that within certain stages of the disease and the age category, the unfavorable prognosis of CC is not influenced by the HPV genotype, number of genotypes, VL, such clinical and morphological characteristics of CC as form of tumor growth, parametrial infiltration, histological type and grade. Along with stage of the disease and age of the patients, HPV-negative status or the presence of HPV 16/18 DNA integration had high prognostic significance regardless of the treatment method. Table 5 shows the results of the predictive model with the optimal discrimination threshold *p* = 0.33. The predictive model has a high statistical significance (*p* = 5.0 × 10^−8^; Nagelkirk’s R2 = 0.336), as well as sensitivity (Se = 0.74) and specificity (Sp = 0.75). Positive predicted value (PPV) = 0.57; negative predicted value (NPV) = 0.86.

The forward stepwise multivariate Cox proportional hazards model showed that the presence of HPV-related biomarker (HR, 6.20; 95% CI, 2.17–17.74; *p* = 0.001), FIGO stage (HR, 3.46; 95% CI, 1.67–7.16; *p* = 0.001), and age (HR, 0.67; 95% CI, 0.49–0.93; *p* = 0.018) remained significant prognostic factors for DFS in all patients.

Thus, based on the results obtained, it is possible with high accuracy to predict clinical outcome (progression) of locally advanced cervical cancer according to the following criteria: HPV DNA status (negative/positive), physical state of HPV 16/18 DNA (episomal/integrated), stage of the disease, age. The application of our model in clinical practice makes it possible to identify CC patients with a high risk of disease progression in order to personalize treatment approaches in the future.

This approach can be used to predict the treatment effectiveness in 78.0% of all patients with locally advanced CC since we were unable to analyze the molecular genetic characteristics and prognostic significance of other (rarer) HPV genotypes of high carcinogenic risk (31, 45, 59, etc.).

## 4. Discussion

HPV status was determined in scrapings from the cervix of 173 CC patients before treatment. The tumors of 20 patients (11.5%) were HPV-negative for 14 high risk genotypes. HPV types 16 or 18 were detected in 115 patients (66.5%). A small proportion of HPV-negative tumors were found in almost all studies using highly sensitive HPV tests. Until now, the question of the existence of true HPV negative CC remains controversial. A number of authors believe that detection of HPV-negative samples can be, at least partially, due to inadequate sampling, limitations in the testing of cytology specimens, deletion or rearrangement of the HPV gene being deleted, low viral load in some cancers, the presence of other HPV genotypes not detected with the current PCR primers and erroneous diagnoses [39,44,45,46,47,48]. The inverse correlation of the number of HPV16/18 DNA copies with the degree of viral DNA integration into the cell genome was revealed in our study. Taking into account this pattern, it can be assumed that one of the reasons for non-detection of HPV DNA may be a very low number of its copies (below the threshold of detection sensitivity) in case of a high degree of integration.

True HPV-negative CC appears to represent a biologically distinct subset of tumors that develop through an HPV-independent pathway and have different sensitivity to treatment as compared with HPV-positive CC. Numerous publications report lower DFS of HPV-negative patients with CC after radiation or chemoradiation therapy regardless of other prognostic factors (age, stage, lymph node metastases) [27,49,50,51]. Our data on the association of HPV negativity with poor prognosis are in good agreement with these findings. At the same time, the clinical outcome in our group of patients with HPV-negative tumors did not differ from that in the group with HPV DNA integrated into the cell genome. These findings support the assumption that at least some of the HPV-negative CC contain a low copy number of integrated HPV DNA, not detectable by the method used.

Our study revealed significant decrease in 5-year DFS in CC patients with integrated HPV 16/18 DNA compared to episomal form regardless of the FIGO stage (II or III) of the disease. A number of authors also report a reduction in DFS of CC patients with stage Ib-IV in cases of HPV DNA integration regardless of the disease stage [37,52,53]. These findings are in good agreement with molecular mechanisms of HPV integration into the cell genome, leading to overexpression of the E6/E7 oncogenes and ultimately to an increase in cancer cell resistance to chemical and radiation exposure [10,11,54].

Contradictory data on the prognostic value of HPV load was observed [29,30,55,56]. Apparently, this discrepancy is due to the fact that VL analysis was carried out, as a rule, in groups including tumors with both episomal and integrated forms of the viral DNA. In these cases, the prognostic evaluation of VL can depend on the proportion of tumors with integrated HPV DNA in the group. 

A single HPV-related biomarker of unfavorable outcome was developed in our study, as DFS of HPV-negative patients was similar to that of HPV 16/18-positive patients with an integrated form of viral DNA. In univariate analysis for patients with stages II-III, OR of unfavorable outcome was 8.9 (95% CI, 2.9–27.6; *p* = 0.0001) in the presence of this biomarker before the treatment. The mathematical algorithm allowing us to predict the clinical outcome of CC patients with high accuracy was developed on the basis of multivariate analysis according to the following criteria: high risk HPV status (negative/positive), physical state of HPV 16/18 DNA (episomal/integrated), stage of the disease, and age. As shown in numerous studies, FIGO stage is the well-known and widely used predictor of clinical outcome in CC patients, taking into account the size of the primary tumor, the state of the lymph nodes, infiltration of the parametrium, and the depth of stromal invasion [2,57,58,59,60]. The age of patients is recognized as a predictive factor in many studies [61,62]. However, a multivariate analysis of the entire complex of possible predictors studied in our work (HPV-positive/negative status, mono/multiple infection in HPV16/18-positive cases, viral load, presence/absence of HPV 16/18 DNA integration, age of patients, stage of disease, histological type, form of tumor growth, presence/absence of parametrial infiltration, the status of the lymph nodes) has not been presented in the available literature.

In accordance with the developed model, the most favorable prognosis of clinical outcome is observed in CC patients with episomal form of HPV16/18 DNA, stage II and aged over 65 years old. The most unfavorable prognosis is associated with HPV-negativity or integrated form of HPV16/18 DNA, stage III and age up to 30 years. The latter group of patients with high risk of poor outcome may require more aggressive therapy and careful follow-up for recurrence [63].

It should be noted that numerous HPV tests and products have been developed in the world and successfully utilized for detecting HPV DNA integration into the cell genome [64,65,66,67]. Potentially, they can be used to identify prognostically unfavorable cases. Currently, there is accumulating evidence of the effectiveness of hyperthermia, as well as immunotherapeutic and targeted drugs in the treatment of advanced and recurrent CC [63,68,69,70,71]. These approaches can be especially effective for the treatment of patients with locally advanced CC who have a poor prognosis according to our algorithm. In addition, highly effective and low-toxicity radiosensitizers including small molecules, macromolecules (such as miRNAs, proteins, peptides, oligonucleotides), and nanomaterials (especially gold-based nanoparticles) can be used to improve the treatment effectiveness in this category of patients [72,73,74].

Thus, the developed prognostic model makes it possible to personalize the treatment of HPV-negative and HPV 16/18- positive patients with locally advanced CC in the future. Personalization strategies can include, on the one hand, the appointment of additional means of radio modification, immuno- and targeted therapy along with cytostatic drugs in combined chemo- and radiation therapy for prognostically unfavorable cases, and, on the other hand, de-intensification of chemoradiotherapy with reducing of treatment-related toxicity in prognostically favorable cases. Our model applies to the majority (approximately 78%) of patients with locally advanced CC. The rest of the patients were found to have more rare genotypes of high risk HPV. Further research is needed to elucidate the prognostic value of HPV DNA integration and other molecular parameters of such HPV genotypes. The relevance of this issue will, apparently, increase over time during large-scale HPV vaccination, which is expected to reduce the prevalence of the more aggressive HPV genotypes 16 and 18 in cervical cancer.

## Figures and Tables

**Table 1:** Clinical and morphological characteristics of patients with CC (*n* = 135).

Clinical and Morphological Characteristics of Patients with CC	Number of Patients (%)
Age, years	
<30	6 (4.4)
30–44	44 (32.6)
45–55	45 (33.3)
56–65	30 (22.2)
>65	10 (7.5)
Stage of the disease (FIGO)	
II	62 (45.9)
III	73 (54.1)
Histological type	
squamous cell carcinoma	119 (88.1)
adenocarcinoma	11 (8.2)
adenocarcinoma with mixed subtypes	5 (3.7)
Grade	
low	26 (19.3)
intermediate	25 (18.5)
high	83 (62.2)
Form of tumor growth	
exophytic	16 (11.8)
endophytic	37 (27.4)
mixed	82 (60.8)
Infiltration of parametrium	
absence(T1b2, T2a)	49 (36.3)
presence (T2b, T3b)	86 (63.7)
Metastases in regional lymph nodes	
absence(T1b2-3N0M0)	65 (63.0)
presence(T1b2-3N1M0)	50 (37.0)

**Table 2:** Molecular genetic parameters of HPV infection in 115 HPV16/18-positive patients with FIGO II-III stages.

Molecular Genetic Parameters of HPV Infection	Number of Patients (%)
Genotypes	
HPV16	95 (63.3)
HPV18	20 (36.7)
Number of genotypes	
mono infection (HPV 16 or 18)	102 (88.7)
multiple infection (HPV16, 18, 31, 45, and other types)	13 (11.3)
Viral load	
lgE7 < 3	4 (3.5)
3 ≤ lgE7 < 5	23 (20.0)
lgE7 ≥ 5	88 (76.5)
Physical state of viral DNA	
absence of integration (episomal form)	49 (42.6)
partial or complete integration (integrated form)	66 (57.4)
Integration degree, *n* = 66	
<50%	20 (30.3)
≥50%	46 (69.7)

**Table 3:** Results of univariate analysis of clinical outcome according to candidate predictor variables (molecular genetic parameters of HPV, clinical and morphological indicators, methods of treatment).

Variables		Outcome of the Disease		OR (95% CI) *p*-Value
		Favorable, Patient Number (%)	Unfavorable, Patient Number (%)	
HPV status	HPV 16/18-positivity	84 (73.0)	31 (27.0)	3.31 (1.23–8.93) *p* = 0.018
	HPV-negativity	9 (45.0)	11 (55.0)	
Physical state of HPV 16/18 DNA	Episomal form	45 (91.8)	4 (8.2)	3.66 (1.96–6.83) *p*= 4.7 × 10^−5^
	Integrated form	39 (59.1)	27 (40.9)	
Genotype	16	72 (75.8)	23 (24.2)	2.09 (0.74–5.85) *p* = 0.170
	18	12 (60.0)	8 (40.0)	
Viral load	lgE7 < 3	3 (75.0)	1 (25.0)	0.90 (0.41–1.97) *p* = 0.793
	3 ≤ lgE7 < 5	16 (69.6)	7 (30.4)	
	lgE7 ≥ 5	65 (73.9)	23 (26.1)	
Number of genotypes	Mono infection	75 (73.5)	27 (26.5)	1.23 (0.34–4.45) *p* = 0.745
	Multiple infection	9 (69.2)	4 (30.8)	
Age category (years)	<30	3 (50.0)	3 (50.0)	0.75 (0.52–1.10) *p* = 0.137
	30–44	27 (61.4)	17 (38.6)	
	45–55	32 (71.1)	13 (28.9)	
	56–65	25 (83.3)	5 (16.7)	
	>65	4 (40.0)	6 (60.0)	
Stage of the disease	II	51 (83.6)	10 (16.4)	3.89 (1.62–9.84) *p* = 0.001
	III	42 (56.8)	32 (53.2)	
Histological type	Squamous cell carcinoma	85 (71.4)	34 (28.6)	2.32 (1.06–5.08) *p* = 0.035
	Adenocarcinoma	7 (63.6)	4 (36.4)	
	Adenocarcinoma with mixed subtypes	1 (20.0)	4 (80.0)	
Grade	Low	21 (80.8)	5 (19.2)	1.25 (0.78–2.02) *p* = 0.356
	Intermediate	15 (60.0)	10 (40.0)	
	High	57 (67.9)	27 (32.1)	
Lymph node metastases	N-	63 (74.1)	22 (25.9)	1.91 (0.84–4.29) *p* = 0.123
	N+	30 (60.0)	20 (40.0)	
Parametrial infiltration	Absence	40 (81.6)	9 (18.4)	2.77 (1.17–6.54) *p* = 0.020
	Presence	53 (61.6)	33 (38.4)	
Form of tumor growth	Exophytic	10 (62.5)	6 (37.5)	1.03 (0.61–1.75) *p* = 0.901
	Endophytic	28 (75.7)	9 (24.3)	
	Mixed	55 (67.1)	27 (32.9)	
Method of treatment	RT	38 (65.5)	20 (34.5)	0.88 (0.49–1.59) *p* = 0.682
	CCRT	48 (72.7)	18 (27.3)	
	NACT + RT	7 (63.6)	4 (36.4)	

**Table 4:** Results of multivariate analysis of clinical outcome of the disease.

Predictor	b *	SE	*p*-Value	OR = exp(b) (95% CI)
Presence/absence of the biomarker	2.269	0.590	1.2 × 10^−4^	9.67 (3.04−30.75)
Stage of disease	1.546	0.471	0.001	4.69 (1.86−11.81)
Patient’s age	−0.497	0.222	0.025	0.61 (0.39−0.94)
Constant	−7.350	1.701	1.6 × 10^−4^	0.001

* b—coefficient of the logistic regression equation.

**Table 5:** Prognostic value of the model for predicting clinical outcome in CC patients.

Observed Cases	Predicted Cases		Percentage of Correct Cases
	Favorable Outcome	Unfavorable Outcome	
Favorable outcome	70	23	75.3
Unfavorable outcome	11	31	73.8
Overall percentage			74.8

## Data Availability

The data that support the findings of this study are available from the corresponding author upon reasonable request.
